# Sphingolipid Metabolism Links Morphological Signatures to Immunomodulatory Potency in Human Mesenchymal Stromal Cells

**DOI:** 10.1007/s12195-026-00933-x

**Published:** 2026-08-27

**Authors:** Daniel C. Shah, Bobby W. Leitmann, Priyanka Priyadarshani, S’Dravious A. DeVeaux, Carlos Munoz, Yuwei Zhang, Kejie Rui, Nathan F. Chiappa, Lauren A. Hymel, William York, Molly E. Ogle, Edward A. Botchwey, Luke J. Mortensen

**Affiliations:** 1 The Wallace H. Coulter Department of Biomedical Engineering, Georgia Tech and Emory, Atlanta, GA 30332 USA; 2 Petit Institute of Bioengineering and Biosciences, Georgia Institute of Technology, Atlanta, GA 30332 USA; 3 School of Chemical, Materials, and Biomedical Engineering, University of Georgia, Athens, GA 30602 USA; 4 Regenerative Bioscience Center, Rhodes Center for ADS, University of Georgia, Athens, GA 30602 USA

**Keywords:** morphology, sphingolipids, mesenchymal stromal cells, lipid metabolism

## Abstract

Interest in human mesenchymal stromal cells (MSCs) as an immune therapeutic has been on the increase over the past two decades with preclinical research yielding promising results, but so far only a singular MSC-based immunotherapy has been approved by the U.S. Food and Drug Administration (FDA). A major barrier for MSC clinical translation is the lack of reliable potency metrics and sufficient understanding of the mechanisms of action, as reported by the National Cell Manufacturing Consortium (NCMC). We integrated high-content morphological profiling and targeted sphingolipid analysis across MSC donors exhibiting a range of IFN-γ induced indoleamine-2,3-deoxygenase (IDO) activities to investigate the role of cell membrane components in MSC immunomodulatory function. High- and low IDO potency cells have distinct morphological signatures that are also reflected in the sphingolipid (SL) activity, with low IDO potency cell lines having low sphingomyelinase activity and high IDO potency cell lines having high sphingomyelinase activity. Perturbation of SL metabolism through the addition of exogenous neutral sphingomyelinase not only shifted morphological signatures to mimic those of high potency, but also significantly increased IDO activity within both high and low IDO potency donors. Additionally, these perturbations resulted in increased extracellular vesicle production with rich SL content and a stark reduction of TNF-α following EV treatment of activated macrophages. Together, these findings link morphology features from potent MSCs to sphingolipid remodeling and suggest a tractable strategy to tune MSC immunomodulatory potency.

**Supplementary Information:**

The online version contains supplementary material available at https://doi.org/10.1007/s12195-026-00933-x.

## Introduction

Human mesenchymal stromal cells (MSCs) have shown considerable promise in modulating immune cell function and reducing inflammatory responses both *in vitro* and within preclinical models. This has led to a rapid expansion of clinical trials from 196 by 2010 to 1299 by 2020 (clinicaltrials.gov), yet there has only been a singular successful FDA approved MSC therapy (FDA.gov). While MSC therapies have consistently demonstrated favorable safety profiles, many clinical studies have reported variable efficacy or failed to meet primary endpoints. These outcomes have been attributed to several factors, including donor-to-donor heterogeneity, differences in tissue source and manufacturing processes, inconsistent cell potency, incomplete understanding of mechanisms of action (MOA), and the lack of validated potency assays and critical quality attributes (CQAs) that reliably predict therapeutic performance. Consequently, researchers, industry, and regulatory agencies have increasingly focused on developing mechanistically informed assays and manufacturing strategies that predict and improve product consistency, potency, and clinical reproducibility.

Many of the most advanced clinical applications of MSCs exploit their immunomodulatory function as a primary MOA. MSCs modulate innate and adaptive immune cells through coordinated receptor-mediated interactions, paracrine cytokine secretion, and extracellular vesicle (EVs) signaling. Collectively, these mechanisms have been reported to promote macrophage and dendritic cell polarization toward regulatory and anti-inflammatory phenotypes [34], induce T-cell apoptosis while blocking proliferation [13, 21], and enhance the induction of regulatory T-cells [4, 53]. A major barrier to the clinical translation of MSCs is attributed to their complex MOAs that have made it difficult to identify clearly defined, underlying biological mechanisms that can link MSC phenotype to functional immunomodulatory outputs.

Generally, physical processes associated with cellular functions (e.g. migration, receptor binding, vesicle release, and mitochondrial activation) often produce distinct phenotypic outcomes that alter cell shape, or morphology, and can be observed using imaging techniques. Recently, the regulatory networks and principles that govern physical changes in cell morphology have been increasingly characterized and applied to understand perturbations in cell morphological dynamics. Interestingly, static morphological profiling of singular images has been used as a tool to uncover underlying phenotypic and functional alterations. These methods have been shown to have high parameter detection sensitivity and are able to predict outcomes such as stem cell fate and cell response to drug treatment [29, 30, 51]. In MSCs, morphological phenotypes have been associated with their differentiation into osteoblasts and chondrocytes, as well as directly predictive of their immunosuppressive activity [26, 31, 41]. Klinker and colleagues uncovered distinct MSC morphological signatures in IFN-γ stimulated MSCs correlative to suppression of CD4^+^ and CD8^+^ T-cell activation [26], and more recently our group has identified that morphological features can be predictive of immune functional outcomes like IDO activity [41].

**Fig. 1:**
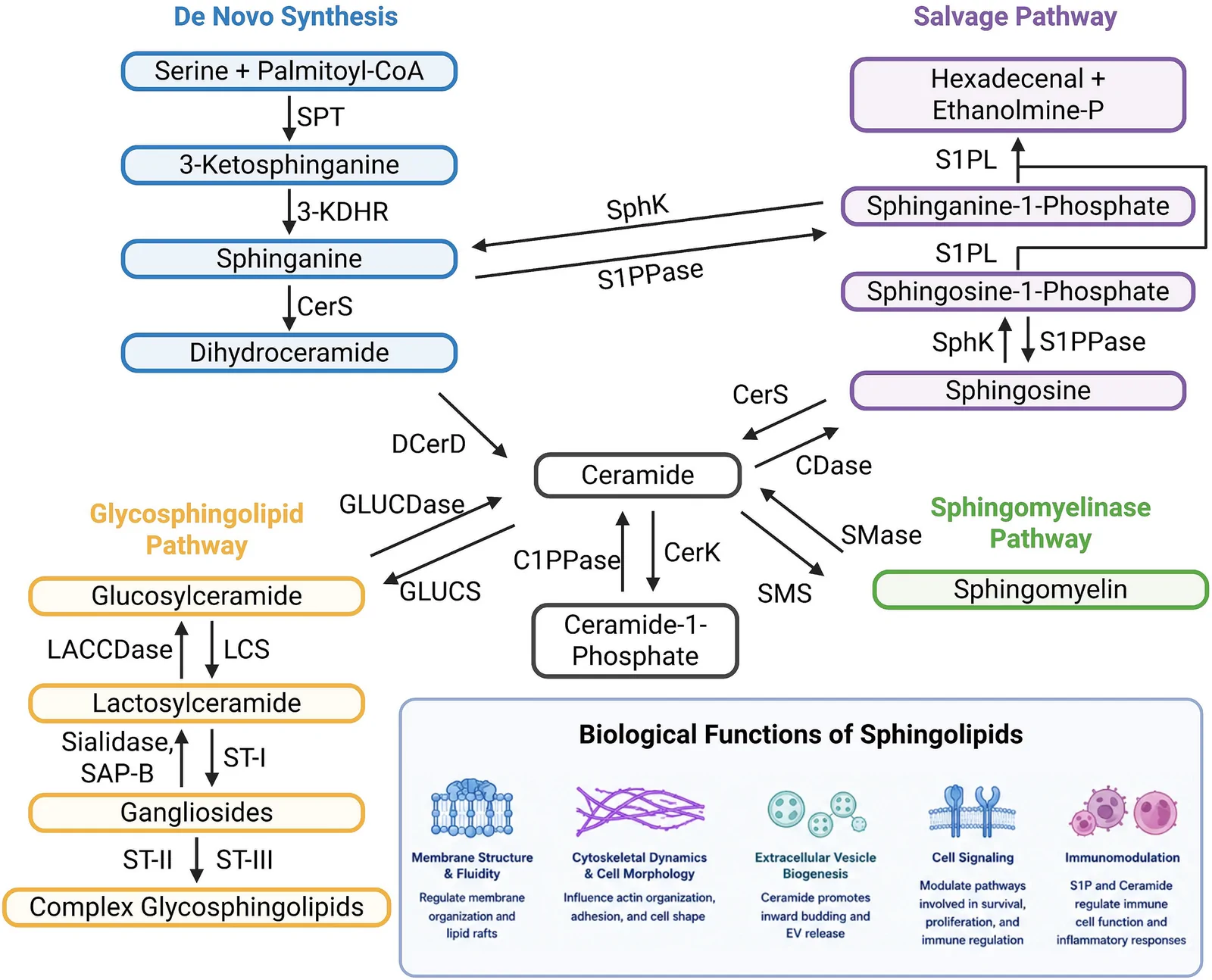
Sphingolipid metabolic network. *Abbreviations* 3-KDHR, 3-ketosphinganine reductase; C1PPase, ceramide-1-phosphate phosphatase; CDase, ceramidase; CerK, ceramide kinase; CerS, ceramide synthase; DCerD, dihydroceramide desaturase; GLUCDase, glucosylceramidase; GLUCS, glucosylceramide synthase; LACCDase, lactosylceramidase; LCS, lactosylceramide synthase; S1PL, sphingosine 1-phosphate lyase; S1PPase, sphingosine-1-phosphate phosphatase; SAP, saposin; SMase, sphingomyelinase; SMS, sphingomyelin synthase; SphK, sphingosine kinase; SPT, serine palmitoyltransferase; ST-I, GM3 synthase; ST-II, GD3 synthase; ST-III, GT3 synthase

Ensemble measurements of cell imaging phenotypes allow inference of cell state and activity, suggestive of underlying network regulation and homeostasis at the genomic, transcriptomic, and metabolomic level [2, 48, 52]. In addition to lipid-associated gene network regulation, plasma membrane (PM) mechanics and their biochemistry are directly related to shifts in cellular morphology with PM lipids serving as critical components of interest during these morphological shifts. Sphingolipids (SLs) in particular play critical structural and signaling roles in regulating actin cytoskeleton and cell shape through adhesive linkages, filopodial/lamellipodial protrusions, and endocytosis/exocytosis processes [12, 16, 22]. Beyond sphingolipid class, the acyl-chain composition of individual SL species also influences membrane organization and signaling. Acyl-chain variants are commonly distinguished by the length and saturation of their N-linked fatty acyl chains; for example, C24:0 ceramide contains a saturated 24-carbon acyl chain, whereas C24:1 ceramide contains a monounsaturated 24-carbon acyl chain. These varying very-long-chain ceramides possess distinct biophysical properties that regulate membrane packing, lipid raft organization, membrane curvature, and protein clustering [23, 46]. Due to the catabolism of sphingomyelin to generate ceramide via sphingomyelinase, alterations in the relative abundance of sphingomyelin and ceramide species can provide insight into changes into membrane and signaling activity within cells [37, 38]. Regulation of the SL network is also closely linked with inflammatory and immune processes, shown through alterations of tolerizing enzymes such as indoleamine 2,3-dioxygenase (IDO), where altered IDO expression has been reported in lipid rich environments [6, 35]. The bioactive signaling lipid sphingosine-1-phosphate (S1P) alters plasma membrane protrusions in stromal cells through the Rho/Rac migratory axis by acting on S1P receptors stimulating migration through S1PR1 or inhibiting migration through S1PR2 [22]. In addition, SL signaling by ceramide and S1P differentially modulates proteins that link actin networks to cell membrane known as the ezrin, radixin, and moesin (ERM) proteins thereby influencing cell adhesion, lamellipodia/filopodia formation, and cell migration, processes modulated in part by the activity of sphingomyelinase [22]. Cytoskeletal and membrane rearrangements required for endocytic invagination and exocytic vesicle secretion are regulated by biophysical and signaling mechanisms that require *de novo* lipid synthesis, neutral ceramidase activity, and sphingomyelinase activity. Mutations that disrupt the balance of the SL network frequently led to defects in inflammatory and immune regulation as well as impairment of skeletal tissues [7, 9, 11, 40, 50]. The SL network (Fig. 1) therefore occupies a unique nexus spanning lipid signaling, cellular biophysics, and cytoskeletal reconfiguration which can be projected through cell morphological properties [32, 33].

We previously used targeted lipidomic profiling to characterize the sphingolipidome of MSCs from bone marrow, adipose, and umbilical cord tissue sources and showed that ceramide, hexosylceramide, and sphinganine species differ between high- and low-IDO-potency bone marrow-derived donors [42]. In this work, we hypothesize that SL profiles could uncover key molecular associations between MSC morphology and immunosuppressive function. We therefore investigated the morphology of several MSC donors with varying immunosuppressive activity and probed correlations between cell morphology and specific lipid-associated cellular compartments. We further examined the SL profiles of MSCs and their responses to IFN-γ stimulation. The identification of key lipid modifying enzymes, such as sphingomyelinase (SMase), was found to be altered in response to IFN-γ stimulation. SMase was then administered exogenously and studied to uncover its role in altering cellular morphology and membrane dynamics. This approach uses imaging to identify underlying metabolic features that can be dynamically tuned to enhance the immunosuppressive function and potency of MSC therapeutic products.

## Materials and Methods

### Cell Culture

Nine donors (RB00037, RB00049, RB00084, RB0114, RB00135, RB00137, RB00139, RB00180, RB00182) of human bone marrow-derived MSCs were obtained from RoosterBio Inc. The MSCs were thawed and expanded once using protocols provided by RoosterBio. Cells were cultured in RoosterNourish (High Performance Media Kit, KT-001) containing serum-derived RoosterBooster under controlled conditions at 37 °C with 5% CO_2_, and appropriate humidity levels. Cells cultured in RoosterBio media or standard hMSC media (α-MEM, 10% FBS, 1% L-glutamate, 0.1% penicillin-streptomycin). If treated with SMase (S7651, Sigma-Aldrich), media was supplemented with 0.1− 0.4 U/mL for 24 hrs.

### Morphological Assessment

Cells were seeded in a 6-well plate at an approximate density of 3000 cells/cm^2^, allowed to adhere overnight, then treated with either 50 ng/mL IFN-γ (SRP3058, Sigma-Aldrich), SMase, SMase inhibitors: 10 µM GW4869 (D1692, Sigma-Aldrich) and 1 µM Amitriptyline (ADVH9C1406F2, Sigma-Aldrich) for an additional 24 hrs. Cells were then fixed with 4% (w/v) paraformaldehyde for 15 mins and washed 3 times with PBS. Fixed cells were then stained with 300 nM DAPI (4′,6-diamidino-2-phenylindole) in 0.1% Tween20 for 10 mins and 20 µM FITC (fluorescein isothiocyanate) for 30 mins. Images were captured with VTI Array Scan High Content Imager (ThermoFisher). Images were then processed using CellProfiler, an open-source software, which extracted 28 morphological features (14 nuclear and 14 cellular features) from the images. These collected nuclear and cellular morphological features consist of: Area, Compactness, Eccentricity, Extent, FormFactor, MajorAxisLength, MaxFeretDiamter, MaximumRadius, MeanRadius, MedianRadius, MinFeretDiameter, MinorAxisLength, Perimeter, and Solidity.

### Lipidomic Sample Preparation and Analysis

Following cell culture as described, samples were harvested and kept at − 80 °C. Samples were thawed on ice and SLs were extracted using previously reported methods [42]. Briefly, each sample was collected for long-chain bases (LCBs) analysis, complex sphingolipids (CSLs) analysis, and total protein quantification (Pierce BCA Protein Assay, Thermo Fisher Scientific) for normalization. LCBs were extracted using methanol:methylene chloride (2:1, v/v), and CSLs using methanol:chloroform (2:1, v/v). Samples were spiked with 50 pmol of internal standard mixture (Avanti Polar Lipids) and incubated overnight at 48 °C. To hydrolyze contaminating glycerophospholipids, 1 M KOH in methanol was added and samples were incubated at 37 °C for 2 hrs, followed by neutralization with glacial acetic acid. For CSLs samples, phase separation was induced by addition of chloroform and deionized water. Following centrifugation (1400 × g, 8 min), supernatants (LCBs) or lower organic phases (CSLs) were collected. Residual pellets were re-extracted, pooled with initial extracts, and organic solvents were removed by vacuum centrifugation (Savant SpeedVac, Thermo Fisher). Prior to LC–MS/MS, LCBs extracts were resuspended in mobile phase A1/B1 (3:2, v/v), and CSLs were extracted in mobile phase A2. Samples were clarified by centrifugation (18,000 × g, 10 min), and supernatants were transferred to autosampler vials. SL analysis was performed on a Micromass Quattro LC system operating in multiple reaction monitoring mode with product ion detection. LCBs were separated on a 2.1 × 150 mm Phenomenex C18 column using a binary gradient (mobile phases A1/B1) at 300 μL/min. The gradient progressed from 100% A1 to 100% B1 over 15 min, followed by re-equilibration. CSLs were separated on a 2.1 × 150 mm Supelcosil NH_2_ column using mobile phases A2/B2 at 300 μL/min with a linear gradient to 100% B2 and subsequent re-equilibration. Lipid abundance was normalized to total protein content.

### IDO Activity Assay

IDO activity was measured as previously described in Daubner *et al.* [17] with minor modifications. Cells were incubated with IFN-γ for 24 hrs; 100 µL conditioned media was collected in round bottom 96-well plate and 50 µL of 30% (w/v) trichloroacetic acid (TCA) was added to each sample to convert N-formyl-kynurenine to its detectable form L-kynurenine. Samples were then centrifuged at 1000 × g for 5 mins. 75 µL of supernatant was transferred to a flat-bottom 96-well plate and 75 µL of 2% (w/v) 4-(dimethylamino)benzaldehyde in acetic acid (Ehrlich’s reagent) was added to each sample. Absorbance was read at 490 nm. Data were normalized by culture media volume, cell number, and days in culture. L-kynurenine standards were prepared in triplicate and diluted with the respective growth media. Representative cell quantification was obtained by PicoGreen. Fixed cells were incubated for 30 mins in PicoGreen reagent buffer and immediately read on a plate reader at 480 nm excitation and 520 nm emission.

### Immunocytochemistry Staining and Imaging

MSCs were seeded onto 96-well plate and allowed to adhere overnight. Cells were subsequently treated with SMase. Following treatment, cells were then fixed with 4% paraformaldehyde for 15 min at room temperature. Samples were then incubated overnight at 4 °C with anit-CD63 antibody diluted 1:100 in PBS. Cells were washed three times using 0.1% Tween-20 in PBS and subsequently stained with CellMas^TM^ Actin Tracking Stain (Invitrogen), diluted 1:1000, for 15 min at room temperature prior to imaging.

### Extracellular Vesicle Isolation and Quantification

Following SMase treatment, conditioned media (CM) was collected and clarified via ultracentrifugation. Briefly, CM was centrifuged at 300 × g for 10 mins, 2000 × g for 30 mins, and 10,000 × g for 30 mins followed by two sequential spins at 100,000 × g for 1.5 hrs, washing with PBS in between. EV pellets were then resuspended in ~ 100 µL of PBS. To measure their size profile and concentration, isolated EVs were diluted 1:100 in PBS and Nanoparticle Tracking Analysis (NTA) was performed using the Nanosight NS300 (Malvern Instruments). Five 60 sec videos were captured with the syringe pump set to 100, camera level set to 13, and screen gain set to 7. Data was analyzed with NTA 3.4 Build 3.4.4 software with detection threshold set to 6. A sample of diluent was also measured and used for background subtraction in downstream data processing.

### PEG-4MAL Hydrogel-Based Cell Culture

PEG-based synthetic hydrogels were synthesized using 4% (w/v) maleimide end-functionalized (four-arm) PEG macromer (PEG-4MAL) (4ARM-PEG-MAL-20K, Laysan Bio), functionalized with a thiol-containing adhesive peptide GCGYGRGDSPG (RGD) (RP20297, GenScript), crosslinked with the protease-degradable peptide VPM (GCRDVPMSMRGGDRCG) (AAPPTec) in 25 mM HEPES (pH ∼ 5.5). RGD and VPM were used at a final concentration of 1.0 mM and 3.3 mM, respectively. 25 µL hydrogels were loaded with approximately 8000 MSCs/gel.

### Macrophage Activation and Flow Cytometry Analysis

RAW 264.7 (TIB-71, ATCC) murine macrophages were seeded at ~ 16,000 cells/cm^2^ and cultured in RPMI 1640 medium (11875093, Gibco^TM^) supplemented with 10% heat inactivated FBS and 1% penicillin-streptomycin. Once ∼ 80% confluent, cells were pretreated with equivalent volumes of EVs for 1 hr before the media was supplemented with 200 µL of 1X brefeldin-A and treated with or without 50 ng/mL of lipopolysaccharide for 2 hrs. Cells were then detached using accutase, pelleted, and fixed in 4% PFA for 20 mins at RT. Samples were washed twice with 1X permeabilization buffer and incubated with permeabilization buffer + anti-TNF-α antibody for 20 mins at 4 °C. Samples were ran on a CytoFLEX (Beckman Coulter) and analyzed with FlowJo.

### Statistical Multivariate Analysis

Statistical analysis was conducted in RStudio (PBC, Boston, MA). A Pearson's correlation heatmap was used to show the differences between high and low potency donors. Morphology feature data was first transformed by computing the average values from a randomly selected group of 30 cells within each condition. This data was analyzed using principal component analysis (PCA) based on the correlation matrix, as the feature scales differed substantially. Graphical illustrations of PCA variable loading plots were generated in RStudio. Partial Least Squares Discriminant Analysis (PLS-DA) was used as a supervised modeling method that incorporated the morphological data to maximize the separation within the groups.

## Results

### Morphological Signatures Differ Across Human MSC Donors

**Fig. 2:**
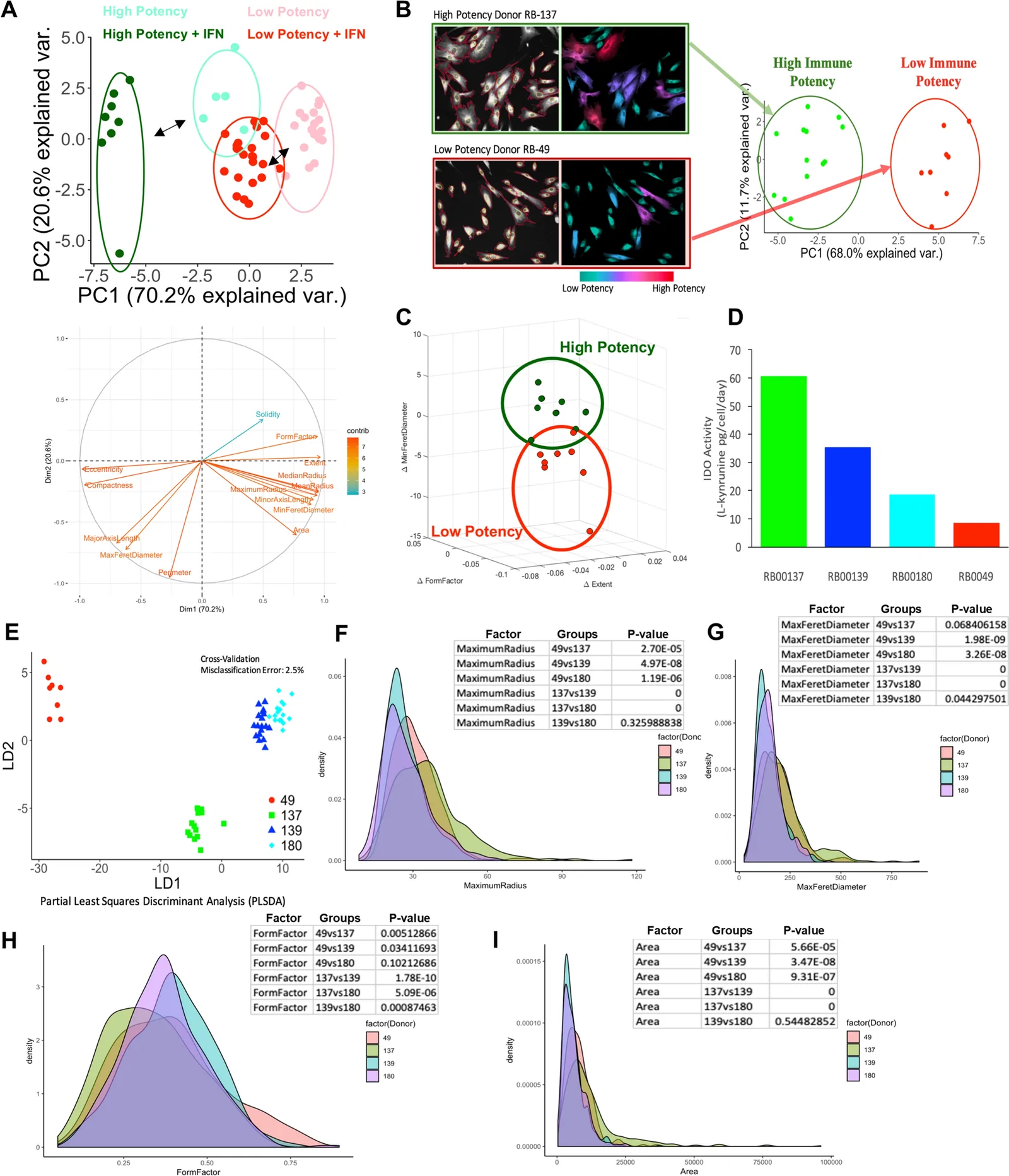
Morphological characterization of MSC immune modulatory potential. **A** Stimulation of MSCs with IFN-γ shows a left shift along PC1 for both high and low potency donors with the high potency donor exhibiting a larger morphological signature change after stimulation compared to the low potency donor. **B** Morphological features of high and low IDO potency MSC donors under basal conditions were obtained with CellProfiler and analyzed by PCA. Morphological features separate high and low potency MSCs along PC1 indicating distinct morphological differences between high and low potency donors. **C** Delta of key features representing ratios of stimulated/unstimulated, selected from highly weighted PC loadings (left) and plotted in 3D show separation between high and low IDO potency donors. **D** IDO activity and **E** PLSDA including intermediate IDO potency donors (RB00139 and RB00180) depicting their IDO secretion and morphological signatures appearing between high and low potency cells. **F–I** Distribution plots reveal differences in morphological distributions across the four donor populations. Statistical significance determined by Kolmogorov–Smirnov test

Differing cellular morphological signatures (as shown in Supplementary Fig. 1A–C) often are used to fingerprint and correlate cellular states, both homeostatic and following stimulus, possibly elucidating information on cellular function. The morphology profile of two MSC donors, one with high IDO potency (RB137) and one with low IDO potency (RB49), were mapped under homeostatic conditions and IFN-γ stimulation. Principal component analysis (PCA) was conducted on measured morphology parameters, with the majority of the variance in the dataset stemming from PC1 (70.2%) and PC2 (20.6%). After IFN-γ stimulation, both the high and low potency donors experienced a left shift along PC1 axis, and as expected, the high potency donor’s morphology signature after IFN-γ stimulation was drastically different than all other conditions (Fig. 2A). Interestingly, we found that under normal cell culture conditions the high IDO potency donor separated robustly from the low IDO potency donor along the PC1 axis. A PC1 loading plot indicated features indicative of cell length and spread (ex: MeanRadius, Max Radius, Extent, FormFactor) were influential in the separation seen along PC1. Using PC1 as an indicator of MSC IDO potency, a color-coded metric was created and cells were overlayed, normalized to the compiled morphology feature measurements. We found that high IDO potency cells tended to have a more heterogeneous population as seen in representative images, while low potency MSCs had a more homogeneous population of small, relatively round cells. In addition, high and low IDO potency cells at basal conditions show distinct separation along PC1 (Fig. 2B). The top three ranked features in PC1 (Extent, FormFactor, MinFeretDiameter) were then used to make differential features derived from the ratio in morphological signatures between stimulated and unstimulated cells and to create a 3D plot of high and low IDO potency cells. A clear separation between high and low IDO potency MSCs can be seen which further indicates distinct morphological signatures for these two donors (Fig. 2C). Two additional bone marrow MSC donors were added (RB139 and RB180), and the IDO activity was measured (Fig. 2D). Morphology of all four donors under growth media conditions was then evaluated and partial least squares discriminant analysis (PLSDA) used to create a supervised multivariate summary of MSC morphology features. We found PLSDA separated each of the donors by IDO potency along the LD2 axis with a cross-validation misclassification error of 2.5%. While RB137 and RB49 (high vs low IDO potency) were clearly separated along LD2, the two intermediate donors (RB180 and RB139) were morphologically similar (Fig. 2E). We then probed density plots (Fig. 2F–I) to investigate the differences in population distributions of morphological features. The Area and MaxRadius features for each donor had significantly different population distributions from each other except donors RB139 and RB180, which is reflected in the population distribution plot. Interestingly, MaxFeretDiameter was similar among RB137 (high potency) and RB49 (low potency) compared to the two intermediate IDO potency donors. FormFactor had significantly different population distributions from each donor excepting RB49 and RB180. The differences found between donors based on population distribution curves further support their distinctive morphological signatures.

### Lipidomic Profiles Reveal Sphingolipid Activity Differences in High versus Low IDO Potency Donors

**Fig. 3:**
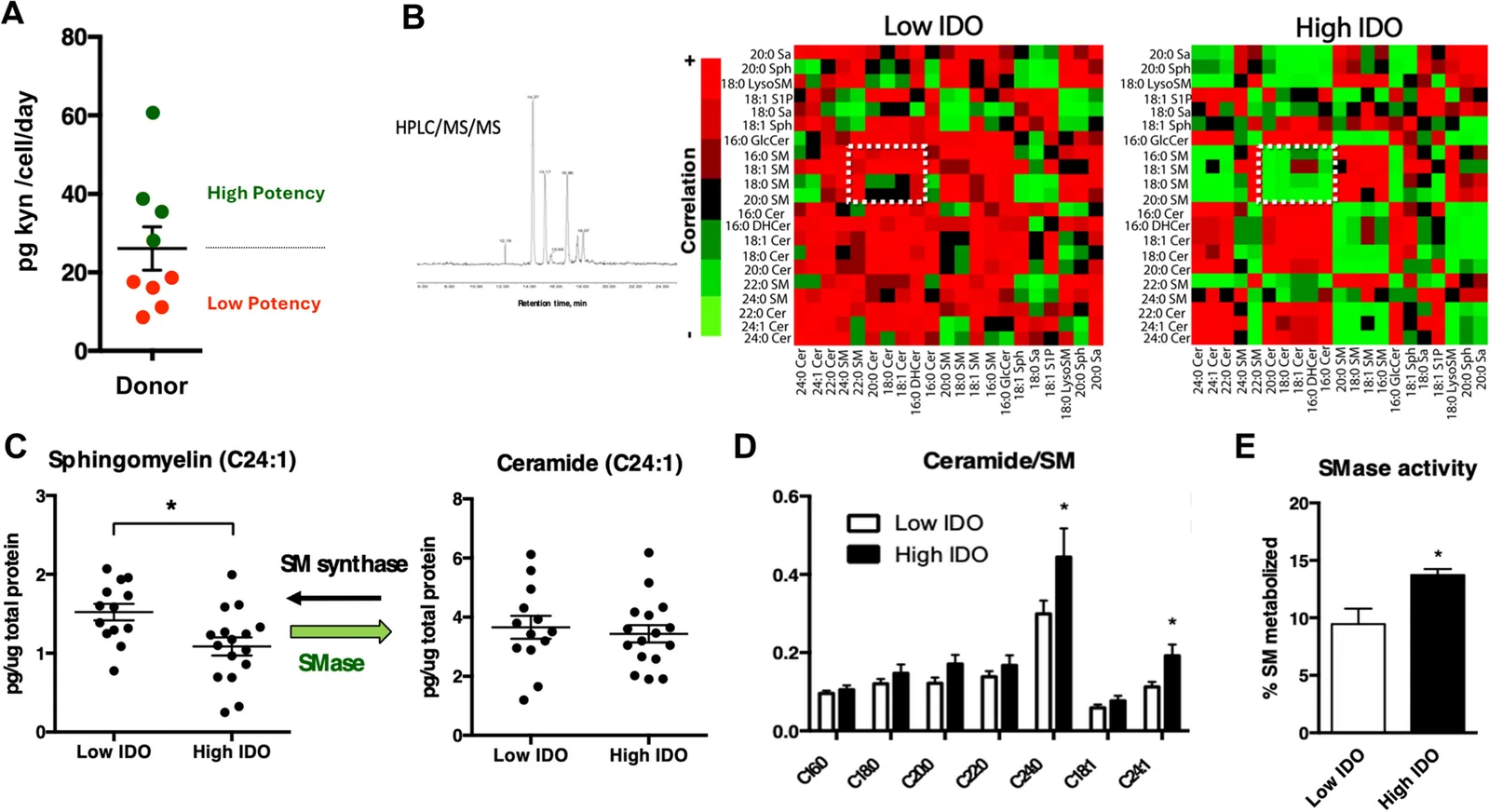
Lipidomic analysis of membrane components reveals sphingomyelinase as a key factor distinguishing high and low IDO potency donors. **A** Donors were grouped into high and low IDO potency using the mean kynurenine production value as a threshold. **B** LC-MS/MS acquisition of lipidomic data was plotted by a Pearson's correlation heatmap showing distinct differences between singular high and low potency donors with lipid annotations plotted on the x-axis and y-axis. Callouts are made to regions with alterations in the relationships between SM and Cer; n = 13-16. **C** Level of C24:1 SM and Cer in low and high potency cells. **D** Cer/SM ratios for different ceramide and sphingomyelin chain lengths in high and low potency donors, n = 13-16. **E** Sphingomyelinase activity is greater in high IDO potency donors; n = 3. Data presented as mean ± SEM. Statistical significance determined by unpaired *t*-tests, *p < 0.05

MSC donors were split into two groups (high vs low IDO potency) for lipidomic analysis using the mean of IDO secretion as the threshold (Fig. 3A). Lipidomic analysis was conducted using high-performance liquid chromatography-tandem mass spectrometry (HPLC-MS/MS) to profile eight donors. A Pearson’s correlation heatmap was generated to assess differences in lipidomic metabolism between high and low potency donors (Fig. 3B). Several key differences emerged between the two groups, most notably in the relationship between ceramides (Cer) and sphingomyelins (SM). In the high IDO potency cells, Cer and SM showed a negative correlation, suggesting active interconversion between these metabolites. In low IDO potency cells, SM and Cer showed a positive correlation, suggesting limited interconversion. Concentrations of C24:1 Cer showed no difference between low and high IDO potency groups; however, C24:1 SM levels were significantly different between high and low potency groups, indicating that high IDO potency groups metabolized SM to a greater extent than low IDO potency cells (Fig. 3C). The ratio between Cer and SM showed that the C24:0 Cer/SM ratio and C24:1 Cer/SM ratio were significantly different between high and low potency groups; further indicating that the conversion of long chain complex SM to Cer was substantially higher in high IDO potency cells than in low IDO potency cells (Fig. 3D). An NBD tracer assay tracking the conversion of SM to Cer as an indicator of sphingomyelinase (SMase) activity and confirmed SMase activity was significantly higher in high-IDO compared to low-IDO-potency cells, with evidence of active SM-to-Cer conversion in high IDO cells (Fig. 3E).

### Perturbation of the Sphingolipid Pathway via Sphingomyelinase Shifts Morphology and Increases MSC IDO Potency

**Fig. 4:**
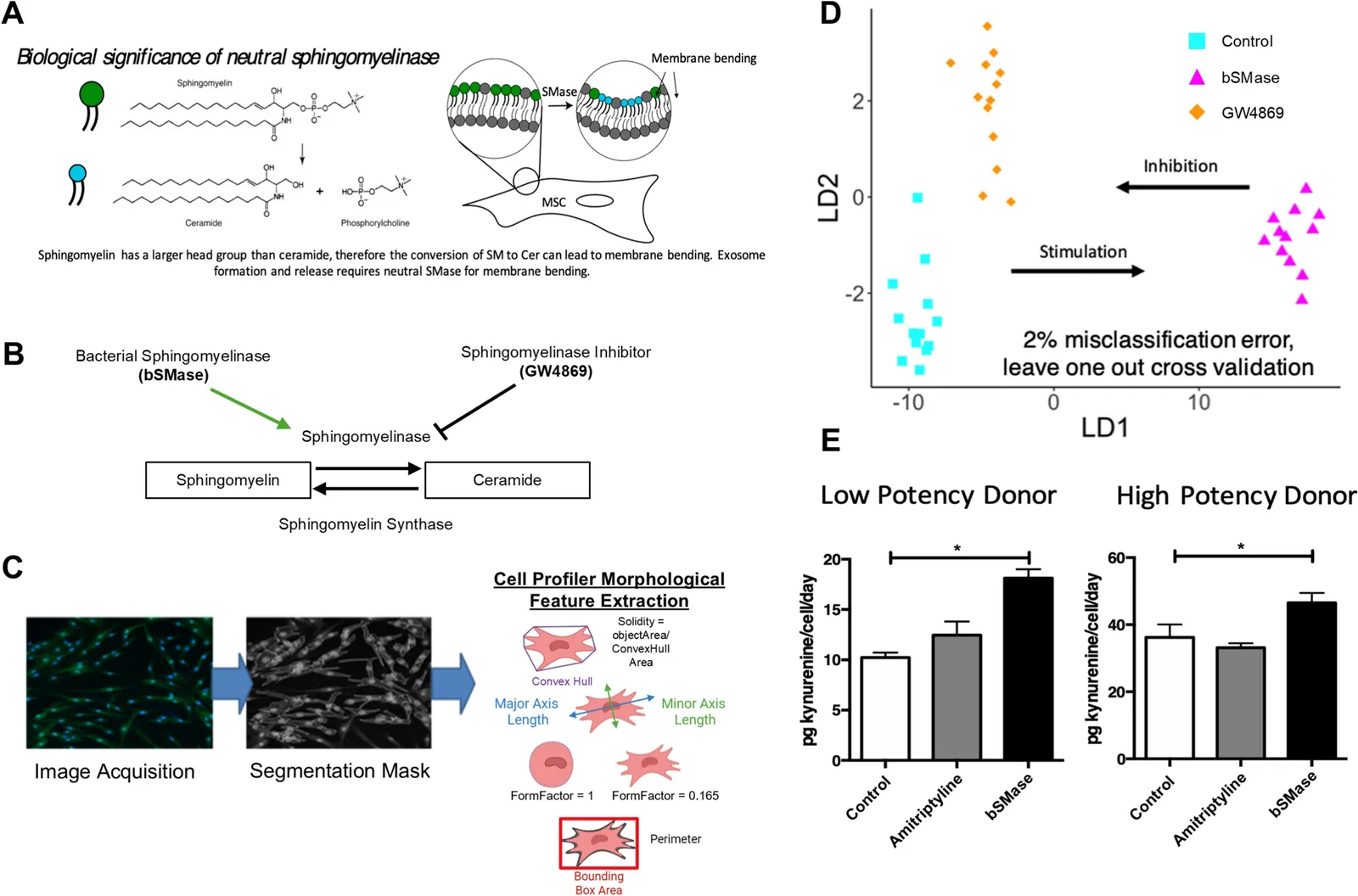
Morphological analysis uncovers distinct profiles of MSC treated with exogenous sphingomyelinase. **A** Bacterial-derived neutral SMase leads to membrane invagination due to **B** metabolism of SM to Cer. **C** Workflow of morphological screening and processing. **D** Treatment with bSMase shifted MSC morphological phenotype relative to control. **E** Treatment of MSCs with bSMase increases IDO activity in both high and low potency donors, n = 3. Data presented as mean ± SEM. Statistical significance determined by unpaired t-tests, *p < 0.05

In this work, we hypothesize that SLs have important roles in both MSC immunomodulation and morphological phenotype (Fig. 4A). Pharmacological inhibitors such as amitriptyline and GW4869 target specific optimally functioning SMase proteins: acid SMase (optimal function at 5.0 pH) and neutral SMase (optimal function at 7.4 pH), and attenuate their enzymatic function (Fig. 4B). We first directly tested the effects of SL modulation on MSC morphology by treating MSCs with bacterial-derived SMase (bSMase), SMase inhibitor (GW4869), or leaving them under basal conditions (control) while morphological features were extracted and compared across all groups (Figure 4C). PLSDA analysis of morphological signatures showed cells stimulated with bSMase had a distinct shift in morphological phenotype compared to control along the LD1 axis (Figure 4D). We then evaluated the immune function of the cells and found IDO activity was significantly increased in both high and low IDO potency donors following the addition of bSMase + IFN-γ in comparison to IFN-γ treatment (Figure 4E).

### Treatment with Sphingomyelinase Increases Secretion of Extracellular Vesicles

**Fig. 5:**
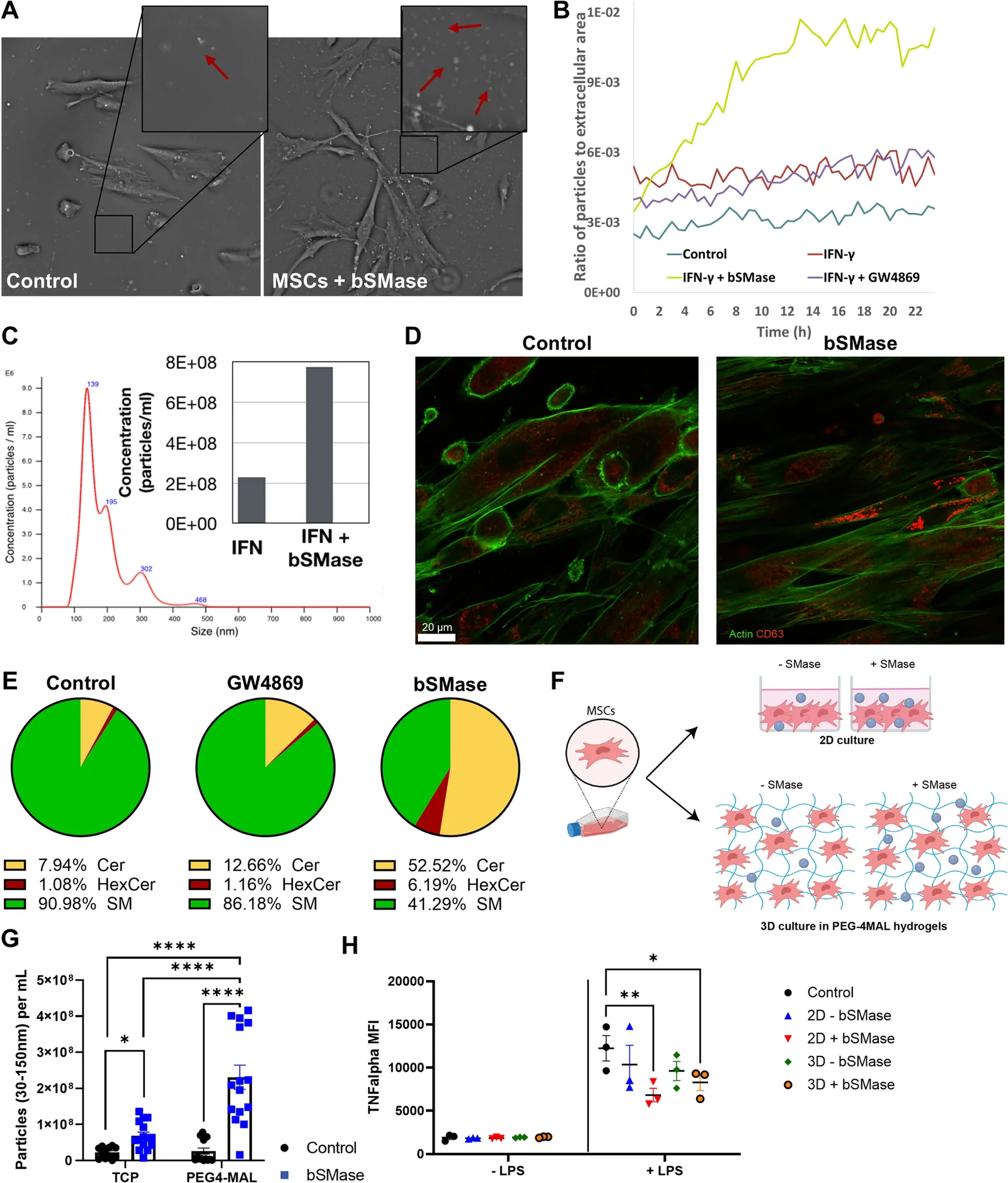
Exogenous bSMase increases extracellular vesicle count in both 2D and 3D MSC cultures. **A, B** Longitudinal phase imaging of MSCs shows an increased number of small particles after stimulation with bSMase. **C** NanoSight quantification of nanoparticles secreted from IFN-γ and IFN-γ + bSMase-treated MSCs. **D** Representative immunofluorescent imaging of MSC labeled for actin (green) and classical endosomal marker CD63 (red). **E** Pie charts of lipidomic analysis conducted on MSC-derived EVs depicting Cer-rich vesicles following bSMase treatment of parent cells. **F** Graphical depiction of 2D (TCP) and 3D (PEG-4MAL hydrogel) EV production. **G** NanoSight characterization of nanoparticle concentration from MSCs cultured in 2D and 3D following bSMase treatment. **H** Intracellular flow cytometry analysis of RAW 264.7 macrophage TNF-α production following LPS activation and/or treatment with EVs produced from 2D and 3D cultured MSCs. Data presented as mean ± SEM. Statistical significance determined by two-way ANOVA with Turkey’s or Dunett’s tests where appropriate, *p < 0.05, **p < 0.01, ****p < 0.0001

Given that treatment with exogenous bSMase enhanced the conversion of SM to Cer in high IDO cells, we sought to determine how this conversion affected MSC functional properties and associated extracellular vesicle dynamics. We treated the cells with bSMase and performed longitudinal high-content imaging. Analysis of these images showed increased secretion of small particles in bSMase-treated cells (Fig. 5A). As IFN-γ has been shown to enhance the functional properties of MSCs, we treated the cells with a combination of IFN-γ + bSMase and compared the release of EVs with other conditions, including IFN-γ alone, IFN-γ + GW4869, and control. The release of EVs was shown to be higher in the IFN-γ + bSMase group compared to all other groups (Fig. 5B). Moreover, there was also increased concentration of small particles in IFN-γ + bSMase group compared to the only IFN-γ group (Fig. 5C). To further demonstrate the increased presence of EVs, we stained MSCs with anti-CD63. The images stained showed higher concentration of CD63 in bSMase treated vs untreated group (Fig. 5D). Lipidomic assessment of isolated control, GW4869, and bSMase-treated MSC-derived EVs was conducted, resulting in identification of Cer-rich EVs following bSMase treatment. The use of exogenous bSMase shifted the sphingolipidome of MSCs to primarily consist of Cer (52.52%) compared to the control (7.94%) and GW4869-treated samples (12.66%), with constant decreased alterations in SM proportions found in bSMase samples (41.29%) compared to control (90.98%) and GW4869-treated samples (86.18%) (Fig. 5E). To understand how these membrane alterations differ in 3D cultures, MSCs were encapsulated in PEG-4MAL hydrogels (3D condition) and stimulated with bSMase (Fig. 5F). Resultant nanoparticle quantification yielded increased concentration in particle concentration following bSMase stimulation in both TCP and PEG-4MAL cultured MSCs (Fig. 5G). Vesicles isolated from both 2D and 3D were distributed to RAW 264.7 macrophages following treatment with or without the inflammation activator, lipopolysaccharide (LPS). Flow cytometry analysis of TNF-α production was conducted and quantified (Figure 5H) uncovering significant reduction in TNF-α levels within 2D + bSMase and 3D + bSMase groups compared to control (LPS only).

## Discussion

The development of robust, scalable MSC therapies requires the identification of attributes that can both predict and control functional potency across donors and manufacturing platforms. Despite extensive clinical investigation, MSC products continue to exhibit substantial donor-to-donor and batch-to-batch variability, complicating comparability, release criteria, and regulatory standardization. A central challenge has been the absence of quantitative, upstream markers that reflect the integrated cellular state responsible for immunomodulatory function. Our previous work characterized the sphingolipidome of MSCs sourced from various tissue types and uncovered altered SL profiles in MSCs contingent of IFN-γ stimulation [42]. In this study, we demonstrate that static morphological profiling captures donor-specific immunomodulatory potential and that SL metabolism, specifically SMase activity, links membrane biochemistry, cytoskeletal organization, EV dynamics, and IDO potency. By integrating high-content imaging with targeted lipidomics we identified a lipid-regulated morphology–function axis in which membrane remodeling acts as a potential upstream determinant of MSC therapeutic potency. This framework suggests that morphological signatures are not merely descriptive phenotypes but quantifiable readouts of underlying metabolic and biophysical regulation. Importantly, SL pathway activity emerged as both a potential discriminative biomarker and tunable process parameter, providing a potential strategy to enhance product consistency and define critical quality attributes (CQAs) for MSC biomanufacturing.

High content imaging of morphological phenotypes has been used broadly to infer various cell states and functions including apoptosis, cell motility, paracrine signaling, contact mediated cell-to-cell communication, mitosis, adhesion, and function [5, 14, 43, 47, 54, 55]. Increasingly, this powerful technique has found application in predicting cell state and functional outcomes in various cell types and pathologies. In a drug-induced liver injury model, Lejal *et. al.* [29] demonstrated that alterations in cellular morphology were predictive of hepatotoxicity and correlated with gene expression programs associated with tissue damage, oxidative stress, and apoptosis, highlighting morphology as an early indicator of cellular dysfunction. Likewise, Way *et. al.* [51] showed that high-content morphological profiling and transcriptomic analyses provide complementary information for mapping cellular state, with image-derived features accurately predicting gene expression programs related to metabolism, proliferation, and stress responses. Expanding on these notions, Alizadeh *et al.* [2] demonstrated that quantitative image-derived features accurately predicted molecular and functional cancer cell states, reinforcing that morphology reflects underlying biological programs beyond simply cell shape. Wen *et. al.* [52] further established a bidirectional relationship between imaging phenotypes and transcriptional state, demonstrating that gene expression can be inferred from morphology and vice versa, thereby supporting the concept that cell morphology represents an integrated readout of underlying molecular regulation.

It is widely known that MSC potency varies depending on the multiple factors including donor and tissue source [25, 26, 28], however differences in morphology may confer a more accurate means to identify potent populations of interest. Several groups have applied these methods to MSCs; reporting morphology is predictive of both MSC differentiation potential and immunomodulatory potency [26, 30]. Klinker *et. al.* [26] found that potency, following IFN-γ stimulation, could be derived from MSC morphological signatures to discriminate between MSC immunosuppressive capabilities. IDO activity in MSCs has more recently been adopted as a gold standard for functional prediction of immune potency [21]. Our recent work has identified label-free morphological markers that are predictive of MSC immune function including IDO activity and a more comprehensive panel of immune function that also included T-cell suppression outcomes [41]. Several groups have reported the role of IDO-mediated MSC MOA through inhibiting IDO using 1-methyl-tryptophan, suppressing MSC immunoregulation of activated Peripheral Blood Mononuclear Cells (PBMCs) [13, 21]. We show MSC donors with distinct IDO activity exhibit separable morphological signatures under basal conditions. Dimensionality reduction techniques revealed cell spread, elongation, and other geometric descriptors strongly contributed to donor stratification, clustering morphological profiles that tended to be by high and low IDO production. MSC immunomodulation function is known to be activated upon stimulation by pro-inflammatory cytokines such as IFN-γ. IFN-γ activates the JAK pathway leading to phosphorylation of STAT1 and the subsequent nuclear translocation for transcription associated roles affecting cellular function [18]. Conversely, STAT1 dephosphorylation has been thought to be mediated by adhesion and cell shape through proteins in the actin cytoskeleton and cellular membrane [10]. This relationship between IFN-γ and cell shape through the JAK/STAT1 pathway could explain the shift in MSC morphology observed following IFN-γ stimulation that is consistent in all MSC IDO potency donors and has also been noted by our previous studies [39, 41] and Klinker *et al*. [26] Morphological screening of IFN-γ stimulated MSCs uncovered a coordinated shift in profiles in both high- and low-potency donors, suggesting that inflammatory licensing drives a reproducible transition in cytoskeletal and membrane states. Interestingly, we found that MSC morphological profiles of high and low IDO potency donors at basal growth conditions were also separable. PCA on morphological features of high and low potency donors under normal culture conditions suggests high and low IDO potency donors could be identified with morphology in the absence of cytokine stimulation. These findings reinforce the concept that morphology could serve as a high-dimensional proxy for integrated cellular states, reflecting possible underlying transcriptional, metabolic, and biomechanical regulation.

To probe metabolomic differences possibly underlying the morphological and phenotypical alterations observed, SLs of high and low potency MSCs were profiled. SLs are known to be instrumental in maintaining the cellular cytoskeleton, shape, and function [27, 32, 33]. These lipids can be concentrated in membrane microdomains known as lipid rafts, which through coordinated membrane organization mechanisms lead to alterations in cell signaling and function [45, 49]. Beyond their structural roles, SLs also play important roles in helping regulate immune responses in elements of the innate immune system, cell survival, anti-inflammatory activity, and homing sites of inflammation/injury [1, 24, 36, 40]. To better understand SL activity within MSCs, 22 SLs were probed within four high and five low IDO potency donors cultured under normal growth conditions. High IDO potency donors had a strong negative correlation between SM and Cer, while low IDO potency donors had a strong positive correlation between these lipids. A study involving lipidomic profiling of MSCs following TNF-α and IFN-γ stimulation identified increased SM levels, which is consistent with our previous single cell lipid profiling data [39], but the specific role of sphingomyelin in MSC function remained unclear, as did potential differences among donors [8]. To dissect the differences between high and low potency MSC donors in relation to ceramide and sphingomyelin, we examined the relative concentrations of differing acyl-chain length Cer and SM in low and high potency donors. The ratios of long chain Cer and SM showed that C24.0 and C24.1 (Cer/SM ratio) were significantly different between high and low potency IDO donors. This suggests that long and complex chain Cer may have specific functions in MSCs related to morphology and immunomodulation and corroborates a paradigm in SL metabolism positing that specific Cer species and their metabolites have distinct bioactive functions in the cell [1, 15]. In addition to Cer and their metabolites, enzymes involved in Cer metabolism such as SMase have direct involvement in regulating Cer production and ultimately their downstream effects. When investigating SMase activity between high and low IDO MSCs, SMase activity was significantly higher in high potency compared to low potency MSCs. The role of SMase in the regulation of immune cell function has been investigated in mononuclear cells, where Al-Rashed *et al.* identified neutral SMase 2 as a regulator of TNF-α induced inflammatory activation [3]. Additionally, induction with SMase was found to be involved in cellular adhesion, reducing integrin mediated adhesion of monocytes through reduced co-localization of lymphocyte function-associated antigen-1 (LFA-1) with ganglioside GM1 enriched microdomains [19].

Given the role of SMase in immune cell function and activation, and the potential insights of high-content imaging of cellular morphology, we probed the relationship between morphology and exogenous bSMase activity in MSCs. The resultant morphological signatures were distinct, clustering parameters in accordance with their treatment groups. Interestingly, inhibition with GW4869 also led to an altered morphological phenotype. Similar findings from Meivar-Levy *et al.* [33] showed inhibition of ceramide production through the *de novo* pathway yielded altered morphological phenotypes in 3T3 fibroblasts. Ceramide synthase was inhibited using mycotoxin fumonisin B_1_ (FB_1_), which altered SL metabolism, affecting lipid homeostasis and actin cytoskeleton maintenance. Given the increased SMase activity in high IDO compared to low IDO potency MSCs, exogenous bSMase treatment of MSCs was conducted to identify changes in IDO secretion in both high and low potency donors. The addition of bSMase to IFN-γ treated MSCs in both high and low IDO potency donors had significantly higher IDO activity when compared to the IFN-γ control. This implicates SMase in having a synergistic stimulatory effect on the immune activity of MSCs. Though the role of SLs and SL enzymes do not yet have a well-defined role in MSC immunomodulation, several studies have explored how SLs affect MSC function, differentiation, and paracrine factor release. Priming MSCs with sphingosine-1-phosphate (S1P) was found to increase MSC mediated cardio-protection in a myocardial infarction mouse model [11]. Others have found S1P treatment in MSCs reduced arterial wall thickness and increased regeneration in a pulmonary artery hypertension in a rat model [24]. In addition to SL players influencing MSC therapeutic function and paracrine composition, endogenous SMase is well understood to be integral in the formation and release of EVs. More recently researchers have uncovered exogenous SLs and their mediators have similar implications in EV formation, often altering their contents and function. Fiorani *et al.* [20] treated embryonic hippocampal cells with Cer and found exogenous Cer facilitated the release of exosomes with a distinct miRNA profile able to influence cellular differentiation in recipient cells. Similarly, we found increased EV formation in both planar and three-dimensional cell culture with additional functional implications in their ability to reduce inflammatory factors within innate immune cell populations. This builds on our previous work identifying the role of exogenous SMase in MSC-EV release and function [44].

Collectively, these findings support the role of sphingolipid metabolism in a central nexus linking membrane biophysics, cytoskeletal organization, vesicle trafficking, and IDO-mediated immunoregulation. Morphological profiling captures this integrated state and provides a scalable, non-destructive proxy for functional potency. By identifying SMase activity as both a putative discriminative biomarker and a tunable metabolic lever, this work advances a structure–function framework for MSC biomanufacturing in which lipid-regulated membrane remodeling serves as an upstream determinant of therapeutic efficacy.

## Limitations of the Study

Although our high-content imaging shows promising associations with MSC potency, the number of MSC donors analyzed was relatively small, particularly in the morphological and multivariate modeling experiments where only four donors were used for PLS-DA classification. While the model demonstrated strong discriminatory performance with a low cross-validation error rate, small sample sizes can increase the risk of overfitting and may limit the generalizability of the predictive framework. Larger donor cohorts will be necessary to validate whether the identified morphological signatures consistently distinguish high and low IDO potency MSC populations across diverse donor sources, ages, and manufacturing conditions. Lipidomic analysis revealed clear differences in sphingolipid composition between potency groups, and the study design primarily establishes correlation rather than direct causation between sphingolipid metabolism and MSC immunomodulatory function. Elevated levels of long-chain and complex ceramides in high-potency donors suggest that sphingolipid pathways may contribute to the observed functional differences; however, lipid metabolism is highly interconnected with other cellular processes, including membrane remodeling, mitochondrial function, and inflammatory signaling. As a result, the observed lipidomic differences may reflect broader metabolic states associated with MSC potency rather than a single causal pathway. In addition, potential immunomodulatory activity was assessed using established surrogate measures, including IFN-γ-induced IDO activity and extracellular vesicle-mediated suppression of LPS-induced TNF-α production, both of which are widely recognized as mechanistically relevant indicators of MSC potency. However, these assays do not directly evaluate MSC-mediated suppression of adaptive immune responses or macrophage polarization. Future studies incorporating orthogonal functional assays, such as MSC co-culture T-cell proliferation or activation assays and macrophage polarization analyses, will be important to confirm that the sphingolipid-mediated morphological and metabolic changes identified here translate into enhanced functional immunomodulatory potency. Together, these limitations highlight the need for larger donor cohorts, mechanistic validation, and expanded functional assays to further define the relationship between sphingolipid metabolism, morphological phenotype, and MSC immunomodulatory potency.

## Supplementary Information

Below is the link to the electronic supplementary material.

Supplementary file1 (DOCX 3988 KB)

## Data Availability

Additional information for further analysis or re-analysis is available through the corresponding authors upon reasonable request. Lipidomic and morphological datasets can be accessed via: https://data.mendeley.com/datasets/ttk3p3h6xc/1
