# Supplementary material for: Sphingolipid Metabolism Links Morphological Signatures to Immunomodulatory Potency in Human Mesenchymal Stromal Cells
Source: Cell Mol Bioeng. 2026 Aug 27;19(4):535–48. doi: 10.1007/s12195-026-00933-x (PMC13562489; doi:10.1007/s12195-026-00933-x)
Supplement: Supplementary file 1 [file 12195_2026_933_MOESM1_ESM.docx]

**Sphingolipid Metabolism Links Morphological Signatures to Immunomodulatory Potency in Human Mesenchymal Stromal Cells**

**Authors:** Daniel C. Shah^a,b,1^, Bobby W. Leitmann^c,d,1^, Priyanka Priyadarshani^c,d^, S'Dravious A. DeVeaux^a,b^, Carlos Munoz^c,d^, Yuwei Zhang^c,d^, Kejie Rui^c,d^, Nathan F. Chiappa^a,b^, Lauren A. Hymel^a,b^, William York^a,b^, Molly E. Ogle^a,b^, Edward A. Botchwey^a,b,*^, Luke J. Mortensen^c,d*^

**Supplementary Figure 1. Morphological differences in MSC population.** Differential phase-contrast image of MSC culture containing distinct morphological differences: (A) large, flat and wide cell (B) small triangular-shaped cell (C) elongated, spindled-shaped cells. Scale bar = 200 µm
